# Current News

**Published:** 1890-11

**Authors:** 


### Current News.

### BANQUET TENDERED TO PROFESSOR WILLOUGHBY B. MILLER, OF BERLIN, BY THE DENTAL PROFESSION OF NEW YORK CITY.

Forty members of the Dental Profession of New York and adjacent cities met at Delmonico's on the evening of October 7, and after an interchange of social amenities, sat down to a banquet that did full credit to that celebrated establishment.

The veterans in professional work were there with the earnest younger men, each anxious to honor their distinguished guest.

After the very delightful menu had been disposed of, the president of the evening, Dr. W. H. Dwinelle, called to order, and in a felicitious address alluded to the work of Professor Miller, and the satisfaction which all felt in having that gentleman in this country, to express to him the deep interest for him personally and regard for his great work, and, in closing, introduced the guest of the evening.

Dr. W. B. Miller expressed his great pleasure in meeting with the dentists of New York and vicinity, and the honor which had been conferred upon him in this and other places had aroused a commingled feeling of thankfulness for the kindness and appreciation manifested, and a wonder whether he was as great a man as his friends were disposed to regard him. These kind attentions, in the West, Philadelphia, and now in New York, would remain a grateful memory, and be an incentive to more continued exertions.

Dr. W. W. Walker then read letters of regret from Dr. H. C. Meriam, Dr. I. L. Williams, and others.

Dr. J. Morgan Howe, being called upon, remarked: While we claim pre-eminence in practice in America, we must give credit to Europe for the work done there. It is pleasant to call to mind the services Professor Miller has performed for our benefit in the investigation and collection of facts during the past ten years. It is undoubtedly true that he has given more of these in regard to dental caries than all the rest of the world in the past decade. In thus acknowledging our indebtedness, we must fully recognize that Professor Miller has made great sacrifices in material things.

*Dr. Frank Abbott.*—I have very great pleasure in being present here this evening to honor our guest. It was original investigation that placed him in an educational institution in a foreign land. Professor Miller, in my judgment, has accomplished more in original research than any one in his particular line of work in the world. We honor all workers. Dr. W. H. Dwinelle, our president, has done more for dentistry in this country than any man now living. How many men could have been drawn together fifty years ago to do honor to any man? I am glad to hear that some time in the future we may have Professor Miller with us in this country, and if this should prove to be the fact, I can assure him of the earnest co-operation of the profession here.

*Dr. C. E. Frances.*—I am supposed to be called upon to make some remarks on our guest's connection with journalism. In the earlier days of the *Independent Practitioner*, Professor Miller contributed largely to it of his original investigations. These were copied everywhere until no one could be unfamiliar with the name of Miller, of Berlin. His labors have been untiring, and have been given gratuitously for the benefit of his profession. I am proud to feel that he is an American, and he can feel assured that if he should at any time conclude to make his home in his native land, it will be with the warm welcome of his professional brethren.

*Dr. W. Jarvie.*—I wish I could represent Brooklyn as it deserves upon this occasion. Many have pointed out the destructive agencies that possibly had to do with caries; but Professor Miller demonstrated the cause, and when the origin is known the remedy can be intelligently applied. We feel our great indebtedness to him; but while we can only show our appreciation at this time, in this way, we would beg him to go on with the work that has given us so much in the past.

*Dr. Norman W. Kingsley.*—I do not know who selected me to represent the profession at large. The remarks that I had elaborated some days ago have gone from me; but I am reminded that "the mills of the gods grind slow, but they grind exceeding fine." I have often wished I could find God's miller, and in his presence, being found, I feel exceedingly small. There are thirty thousand dentists in this country; but there is but one Miller. I don't expect ever to be a Miller. He has carried his investigations to a point that there is no possibility of our ever reaching him; indeed, I may say, I have no desire to attain to this, notwithstanding my ambition is to be in the front rank of the profession. Let us accept his investigations of microbic life as conclusive.

Dr. Kingsley then alluded to the Italian law recently enacted, requiring six years of study in medicine, after which the study of dentistry may be entered upon. It would, he remarked, be a sad day if such an edict were passed in this country. Would it be possible for a man after such a course to attain results reached here? Suppose Thalberg or Josephy were obliged to go through this theoretical training, could they ever have become masters in musical execution without the manipulative skill acquired by the use of the fingers? If such is to be the training we may have scientific men, but how the community will suffer. Shall we speak of ourselves as only dentists? I am only a dentist, and am not ashamed of the name. There is one thing I admire in Professor Miller, and that is he never repudiates dentistry.

*Dr. W. H. Atkinson.*—Brethren, it is no light thing to be esteemed by those who know us well. If there is an individual I reverence, it is a naturalist. There are by-ways in which inspiration flourishes. How many are willing to accept the inspiration of the moment? You refer, Mr. President, to my history. I have no doubt but it might be of value to some. Text-book teaching has been accepted and found false; but when the truth in its simplicity has been discovered, then, each and all know what is meant by it.

Compare this night with 1862, and you will see what the angels of light have done for us. Our brother has simply been delivered from false authority. When a discovery is made, let it be told. When one is converted, he goes forth and proclaims it. Let revivals become perpetual, and then we will have no more quarrels.

*Dr. L. D. Shepard*, Boston.—I have been sitting in fancied security, because I had not been informed that I was expected to speak on this occasion. I am glad, however, to respond to the request. I feel forced to portray to you a character. A young man graduates in a school of learning, goes to Germany, and from there commenced the study of dentistry. The heart became involved in the work, and also the intellect. From a student in the University of Berlin, he eventually was called to fill, by the force of his acquirements, a chair in that same institution. We are here to-night to recognize the work that led to this honor. Those who were present in Berlin, and witnessed the ability and tact which Professor Miller displayed as secretary of the Section of Dentistry of the International Medical Congress, could bear witness that amid many jealousies and conflicting interests, he maintained the respect of foreigners and the people at home.

We think lightly of the labors that have brought about results made familiar to all of us. I stood, in 1883, in his laboratory in Berlin. With all the outer air excluded to prevent the ingress of micro-organisms, I found the atmosphere unbearable, and returned to my home with a violent headache. It must be remembered that Dr. Miller experienced the injurious effects of this condition night after night and month after month, in order that the results might be verified. We should for these great sacrifices do something more than merely applaud. We should combine and make a substantial fund to compensate those who devote their lives to original work.

*Dr. James Truman*, Philadelphia.—While not anticipating a call to speak on this very interesting occasion, I cannot refrain from adding a few words. When at the gathering in Philadelphia last evening, where were assembled the foremost men in medicine, science, and in our own profession, it seemed to me that at last dentistry had been elevated from the ragged waif—the forlorn child of the professions—to the position which of right belonged to it, shoulder to shoulder with its elder brother. When Dr. Harrison Allen, emeritus professor of physiology in the University of Pennsylvania, in response to the toast, "The Bond of Union between Medicine and Dentistry," remarked, "Why, gentlemen, there is no bond, they are one and the same thing," I felt that the demand for recognition, of which we have heard so much, had at last come, and in the only true way, and that so fully illustrated by the guest of the evening, in the cultivation of the best within each one of us.

I have been much impressed with the spontaneity of these efforts to express gratitude for Professor Miller's work. Without collusion, Cincinnati, Buffalo, Philadelphia, and now New York hastened to do him honor, and in a short time our brethren in London will extend, in a similar way, their congratulations and best wishes for an enlarged future. I join with you on this occasion, in the paramount thought of the evening, that our guest may still continue to lead in investigation in the lines he has so happily marked out in the past.

*Dr. E. C. Kirk*, Philadelphia.—One thought has been with me for some time, and when the president requested me to speak, I thought it might serve as an illustration, to give you an allegory. A certain official had a dream, and it appeared to him that he went to the realms of eternal bliss. The applause which he received there for his work was so overwhelming that it created in his mind a strong desire to return to earth and continue the labor that had been so productive in increasing his happiness. The moral of this seems to be that we hope the return of Professor Miller to Germany may result in a similar manner.

During my college days I sat on the same bench with Dr. Miller, and naturally felt a deep interest in him and his work. He was actuated then by a desire for knowledge based on accurate investigations.

We are not here to do honor to Professor Miller. His work does that for him. Let him carry home the feeling that we—his professional brethren—are grateful for the means he has placed in our hands to combat the evils we meet with in practice.

*Dr. C. S. Stockton*, Newark, N. J.—Every person has at some period in his life the desire to look upon the faces of the great world-workers. Now I have had this feeling in regard to our guest. I am glad to be here to-night to represent New Jersey, and to meet him face to face, and to express to him that, in my opinion, no man has done more to raise dentistry to a higher level than has Dr. Miller of Berlin.

*Dr. S. S. Perry*, New York.—For the last twenty-five years there has been a marked cultivation of the mechanical side of dentistry to, perhaps, the exclusion somewhat of the theoretical. Dr. Miller has given us the means of saving teeth other than by mechanical agencies. In dentistry there exists the same pathological conditions as in medicine and surgery. We will, in the future, be obliged to combine the skill of the surgeon with the theoretical knowledge of the general practitioner.

*Dr. C. F. W. Bödecker.*—I have felt very much like the passenger on the boat this evening, who said to the captain, "Let me sail this boat, I can sail it as well as you," but when it came to the test, he failed to hold the boat to the wind. Now, I feel as though I was called upon to sail without bringing my boat into the wind, as it has all been taken from me by the previous speakers. I cannot add anything to the appreciation manifested; but it is felt to the same extent as has been expressed by others. I am proud of Professor Miller's position in Europe; but for myself, I regard this country as my home and possibly with right, as I have been here now twenty-one years, and could not think of giving up my naturalization, as I could not feel as comfortable as I do here.

*Dr. L. S. Straw*, Newburgh N. Y.—I wish I was a Miller; but, not being one, all I can do at this late hour is to extend to our guest my right hand, as an evidence of my sincere appreciation of his work.

Dr. Dwinelle, the president, then requested those assembled to rise and drink to the health, continued prosperity, and successful voyage of our guest to his adopted home.

Thus closed one of the most satisfactory and enjoyable banquets held by the dental profession in New York. The feeling of sincere appreciation manifested doubtless left its impression on the gentleman the banquet was intended to honor, as it certainly did on those present, and its informal character gave to it a charm not always felt upon similar occasions.

---

### BANQUET TO PROFESSOR W. B. MILLER IN PHILADELPHIA, OCTOBER 6, 1890.

The reception in honor of Professor W. B. Miller, at the Colonnade Hotel, on the evening of the 6th of October, was remarkable in many respects, and more worthy of special notice than such occasions usually are. The Committee of Arrangements very wisely determined to make it a gathering of the best men in medicine, dentistry, and general science. The result justified the wisdom of this, and the addresses partook in consequence more of the character of a dignified discussion of principles rather than the ordinary vein of "after-dinner" speeches.

It is with regret that these remarks from representative men cannot be reproduced in full here; the mere abstracts we have space for do but feeble justice to them. They were, however, stenographically reported, and will in due time appear in proper form.

Professor E. T. Darby, in calling attention, as president of the evening, to the presence of the distinguished guest, remarked:

"About thirteen years ago a young American gentleman, residing abroad, came to me with a letter of introduction. This letter stated that the bearer was about going to America to pursue his studies in dentistry, and that he gave promise of being an ornament to the profession he had chosen. The gentleman introduced is our guest this evening; the one who wrote the letter is also present, and I shall ask him to respond to the toast of 'Our Guest.' Professor James Truman, of the University of Pennsylvania."

Professor James Truman, in response to the toast, called attention to the marks of progress that had been made in scientific history, in this particular research, from Leeuwenhoek to Pasteur, from Pasteur to Koch, and from Koch to Miller. After alluding to the men of the past and those who surrounded him, distinguished in various walks of science, he discussed for a few moments the tendency of the age to seek special lines of work, and the effect of this upon the person and upon the State. He then alluded personally to Professor Miller, and said, "I remember very well, some thirteen years ago, Professor Miller, then a student in Berlin, calling upon me while in that city, and requesting my advice as to the selection of a profession. Whether the advice I gave him at that time was taken or not, he came to America and graduated in dentistry at the University of Pennsylvania.

"It is important to lay a good educational foundation, and, appreciating this, we find our guest starting out in life at the Michigan University; from Michigan he went to Edinburgh, from Edinburgh to Berlin, from Berlin to the University of Pennsylvania, and from there back to Berlin, to receive, in due time, a professorship, the first American so honored."

Allusion was then made to a visit in 1880 to Berlin, and an interview with Professor Miller. He was then busy in his laboratory, and soon thereafter appeared those remarkable papers on dental caries which have made him known throughout the world. He then, in a few brief sentences, on behalf of the Odontological Society, welcomed the guest of the evening to Philadelphia, the Mecca of medical science, and to the city where dentistry has always been honored, and where it has accomplished some of its best work.

### Toast: *The Dental Specialty in Europe.*

Professor W. B. Miller said, "I cannot endeavor to respond to this toast without first expressing to my friend, Dr. Truman, my sincere thanks for the very kind and flattering words which he has just uttered. I wish also to express to the Odontological Society of Pennsylvania and to the representatives of the dental profession here to-night my sincere thanks for the part they have taken in organizing this complimentary dinner, which is so flattering to me. Again I wish to express my thanks to the many eminent members of the medical profession who have not deemed it unworthy of the occasion to be present. I can assure you that I recall the fact that many of you here to-night were distinguished in your separate professions before I thought of studying dentistry or anything else that I can recollect. When I recall to mind that I was a student under many present, I feel unworthy of the honor which you are paying me, and will rejoice if I shall ever be able to feel in the future that I am to some extent at least worthy of it.

"In regard to the question of the dental profession in Europe, the practice is so different in the various states of Europe that it would be impossible, in so short a time, to give any idea how it is carried out in each. The dental profession in Germany is in a peculiar position. In 1889 a law was enacted which is called *Gewerbe-freiheit* (freedom of trade, freedom of profession), conferring upon every one the privilege to practise medicine or any specialty of medicine without any qualification whatever. The effect of this law was not marked upon the medical profession either for or against. In regard to the dental, it had, however, a serious result. After its enactment, a class of practitioners arose in the practice of dentistry without any qualifications. To illustrate the effect, I will give you an example: A distinguished physician had a married woman to attend to his office, to see to the proper cleansing of the instruments, adjusting the rubber dam, etc. Her husband was the servant of another dentist, and he, being in the mechanical laboratory of his employer, acquired some knowledge of artificial work. In course of time they established themselves in a dental office without having attended any school, or having received any education worthy of mention. The lady took in hand the extraction of teeth and the husband the mechanical part. In a short time they had a lucrative practice, numbering among their patients a duchess. This will explain the large increase of this class. While there are at present not more than one thousand legally-authorized dentists, there are four or five times as many in the practice of dentistry who have no qualifications, except that they have picked up the information as chance offered. These men are sometimes skilful, and they are able to compete with those who have attended college two years and have passed the examinations. It was but a few years ago that dental students in Germany received any instruction in practical dentistry, and consequently the dental practitioner was generally launched upon the world without sufficient knowledge. It will, therefore, be understood why 'teeth artists' and 'teeth mechanics' were able to enter into competition with the regular dentists. This has had a certain good effect, because the regular dentists are beginning to recognize the fact that they are not able to compete with this number of 'teeth mechanics,' and are beginning to better qualify themselves for dental work. In the last few years marked progress has been made in Germany. Years ago a few dentists went over to Germany to practise dentistry, and exerted a marked influence. The Germans caught on to their methods, and have now attained to a high standard of practice. Within the last six years the first schools of dentistry have been established as departments of the various universities in Germany. At first there were only two, one at Berlin and the other at Leipsic. Since the dental school in Berlin has been established a large number of students have studied in that city, and it has been our earnest endeavor to emulate if not to arrive at the same point of excellence as the dental institutes in America. It is not possible in a few years to create a dental college in Germany which can be placed upon the same footing as the older dental colleges in America, but we are doing all we can to acquire this point of efficiency, and we find our students earnest and industrious. In one respect we think our dental institutions are better than those in America, because we engage personally in the instruction of the students in the clinics. I have to be at the dental institution two to three hours daily, giving instruction in dental manipulations, filling teeth, etc.

"I come now to the third point, which I consider of more importance than those to which I have referred, a point which has had more effect upon the development of the dental specialty in Germany than any other, that is the study of bacteriology. As you know, it is but a few years since the introduction of the well-known methods of Professor Koch, by which the study of bacteriology has been made accessible to every one. Many have since engaged in the study in the human mouth. This has been a portion of my work. A great many micro-organisms are present there, and they find a fertile field for vegetation, feeding upon the dead matter therein contained. Up to the present date seventeen micro-organisms, which are divided pathologically into three groups, have been discovered in the human mouth, and have pathogenic properties. When we consider the fact that in the oral cavity not less than seventeen pathogenic micro-organisms have been found, and that a larger number may yet be discovered if experiments are continued, we may have an idea of the effect upon the human body. This has created great interest upon the part of physicians as well as dentists, and many clinical cases have been reported which bear out the fact that the oral cavity exerts an influence upon the general health, and that the profession of the dentist is second to none in importance. I will give you a few instances to substantiate this view. In looking up the literature of the case, I was able to find ten to fifty cases of death resulting from abscess of the teeth or from dental operations which had been carried out under improper antiseptic conditions. Some time ago a student of mine, engaged in similar investigations, was able to find in a short time the history of not less than nineteen cases of similar character, which had occurred in two hospitals in Germany. Some months ago a man was found dead in the neighborhood of Berlin. There were no signs of external violence, no chemical agents had been used to bring on death. The autopsy brought out the fact that death resulted from meningitis, which was traced to caries of the teeth. A few months later a lady graduate in a dental college had the misfortune to wound her hand with a dental-engine bur while operating upon a carious tooth for a patient, and the last I heard of her was that there was little hope of her recovery. There are hundreds of such cases, but the practitioner is not willing to make them public.

"A distinguished specialist of Posen has occupied himself with this subject, and he mentions that loss of appetite, nausea, general ill-health may be brought about by improper attention to the mouth, causing a chronic state of putrefaction, the products being absorbed by the mucous membrane, with serious results to the general health. He was able to restore a patient by nothing more than properly cleansing the mouth. It has also been ascertained that the condition of the oral secretions and caries of the teeth act in other ways. Examinations of nine hundred and eighty-seven children in this way demonstrated that ninety-nine per cent. of all those suffering from caries of the teeth were affected with putrefaction, swelling of the lower glands, etc., of which no physician would be able to make a diagnosis.

"I examined one case from which there was an unpleasant odor to determine how many bacteria may be in the human mouth at one time. I found there were millions of germs. Of these, a large number are swallowed at every meal-time, and in persons predisposed exert a malignant effect upon the general health. I will also cite one significant case which occurred in Berlin. James Israel, who has done so much work in this direction, discovered in one lung a very small body not much larger than the head of a pin. He sent it to me for examination, and I found it was dentine broken off from the root."

Dr. Miller then entered into an explanation of how a certain pathogenic form is a constant inhabitant of the mouth, and entering the lungs may develop pneumonia, and experiments, recently made, have "almost established the fact that micro-organisms may exist in the lungs for a long period without any ill effect." He then continued:

"These facts I think are sufficient to show the immense importance of a proper care of the human mouth, and the action which it exerts on the general health, and we consequently will not be astonished that in Germany there has been a marked progress in the dental profession in the last two years. Physicians have taken more interest in our specialty; at the same time we need a higher standard of education in order to bring out a better appreciation of these facts, and to this end we are aiming in Berlin to increase the course of study. At present we have virtually a three years' course instead of two, which we had a few years ago. At the same time physicians as well as dentists recognize the fact that medicine rests upon common ground with dentistry, and it would be as impossible to separate medicine from dentistry as to separate the human mouth from the alimentary tract.

"I am afraid I have not been able to present this subject to you in the way I wished. I have tried to be brief and say much in a short time, and consequently I have, I fear, not said much of anything. I wish, however, in closing, to repeat my expressions of thanks to you for the honor conferred upon me, and hope that I shall be able to continue my experiments, and if I do not merit the distinction now conferred, which I do not believe I do, I hope I shall be able to merit it at some future time."

TOAST: *Educational Demands of the Healing Art.*

Provost Wm. Pepper, in responding, expressed his thanks to the committee for the privilege of hearing the statement, most interesting and important, made by our distinguished guest. He then desired to call attention to several subjects from the stand-point which he naturally would consider them, and then remarked:

"I think we can take credit to ourselves in this country,—and in this city particularly,—for the pioneer way in which we frankly recognized the dental profession as an integral part of the great medical profession, the dental practitioner as the colleague of the medical practitioner. In this way we contribute powerfully to promote dental science, to strengthen dental institutions, and to encourage dental practices in the presence of a certain prejudice which the younger men present will scarcely appreciate, but which we older men can remember. I am glad to be allowed to speak on this subject, as being connected with an institution that I am proud to say was prompt to act in this matter. There is no department of the University of Pennsylvania to-day which is regarded by the authorities of that institution—I may say by the community—with greater interest and more worthy pride, none in which the character of the work is more earnest, thorough, and good, in which the standard is more steadily maintained, is higher, and the determination to carry the grade of work to the highest attainable point is more settled and energetic, than in the dental department.

"The 'Educational Demands of the Healing Art,' as it impresses the general practitioner and the specialist, is no doubt a difficult subject. The requirements are becoming higher. I think we all recognize in this country that it is not so much that we have not a high standard, as it is that the conditions under which we have been living for the past century have been inimical to it. In this country, where the conditions are favorable for embodying our ideas in practical form, we find medical men and dentists, and all classes of scientists, are earnest in seeking to establish the highest grade of education that is within their reach. The dental specialty was started in America and placed upon so high a position that the world has been obliged to be its imitators. But, from what we have heard from Professor Miller it seems clear that unless we realize that our educational requirements in the healing art are high and exacting, we shall not, in this specialty, maintain our pre-eminence. We may be sure that the same thorough spirit which has made German medical education an exemplar of all medical institutions of the world, will be applied to the dental specialty, and unless we practitioners in America insist that our educational requirements shall be increased as the capacity of our students coming to us is higher, and better fitted to receive this knowledge, we shall find that in the few points where we have had precedence, we shall begin to lag behind. I feel confident, so strongly have we grasped that truth, the time is fast approaching when we are going to establish our institutions upon a stronger basis and raise their standard.

"Is there not a thought suggested by our guest and the remarkable career he has pursued, and which would render such a career exceedingly difficult in this country? Is not one of the requirements most urgently needed for our education in this country the endowment of research, a position where men of ability could have their maintenance secured to enable them to devote part of their time to abstract study, a part of that time which is with us nearly wholly consumed in practical details? I do not pretend to draw inferences from so remarkable a person as our guest. There are certain persons whose energy and power of aiding themselves are so extraordinary that they accomplish results, although hampered with work, that the rest of men could not accomplish if they devoted their whole time to it. There are three men around this board whose work I know well. Miller is one, Cope is another, and Wood is another, each a pioneer in the most difficult and abstruse subjects; men whose whole talents should have been devoted to the pursuit of these investigations, and yet not one of whom would have found in America, save in the employ of the government, any means to pursue work of this kind, nor do we see, except in a few places, much tendency to meet this endowment so much required. If our education in any branch is to keep pace with the movement of the times, with what is being done abroad, if we are to furnish our quota to this remarkable epoch, we must have practical men come in and apply their principles. If America is to have her share in this, if this educational standard is to be maintained, it is necessary that our institutions shall not only present the highest equipment, but that they shall demand the best teachers, masters of their respective branches, and that they shall possess an endowment which shall give maintenance and a secure home to scientists, men like our guest, who are able to see further and clearer into the difficult problems which are before us, to open paths in which we can walk securely. Unless we can insist on this in our country, America will fall behind. Thus it behooves us to hold high our standard, to be discontent with our past achievements, however fine they may have been, and not lose sight of the fact that when we have secured an advantage we must hold it, Remember that practical success is not of the highest importance; the demonstration of scientific truths constitutes the only real basis for future successful practical work.

"These requirements, Mr. Chairman, have occurred to me as I listened to the statement of Professor Miller, and I feel that we should carry away with us a determination to embody them in our educational work. I feel honored to be present here to-night, and to be able to express with my voice my congratulations of the world-wide reputation which our friend has obtained in a few years, and to wish him 'God-speed' and further triumphs in his researches."

TOAST: *Modern Methods in General and Dental Surgery.*

Professor W. W. Keen, in responding, said he should prefer in one language, as Dr. Wayland said a friend of his did "in seven, keep silence;" but he felt the necessity of expressing a word or two upon an occasion such as this. He then very wittily alluded to the work of Professor Miller in connection with micro-organisms, and then remarked: "He also gives us another illustration that a man cannot know too much, not only in his own specialty, but also in many others which, apparently, are not allied to it. His earlier studies in chemistry, which were so complete, have been his handmaid in his investigations of dental caries, and had he not been an accomplished chemist he could not have performed the experiments he has done. Not only is he a chemist, but an exact observer, a bacteriologist, well versed in pathology, and an eminent dentist. This bacteriological study, and the other allied departments which he has laid under tribute, is precisely in the line of our advances in general study. Bacteriology has revolutionized the theory and practice of dental surgery, and this is an encouraging sign of the times, and is in line with that revolution whose story reads like a dream, and which has passed before our eyes in general surgery."

He then called attention to the advances made in dental surgery in the transplantation and reimplantation of teeth, with results as "gratifying to the patient as to the dentist. . . . The deformities of the mouth are now the playground of the dentist, in which he moulds and turns the jaw to suit the face."

"I cannot," he said, in closing, "too warmly express my own feeling of obligation, in many departments, to our dental brethren."

TOAST: *The Relation of Modern Pathology to Dentistry.*

Professor E. La Place spoke of the "deep appreciation of the kindness" that called upon him to respond to the toast, "The Relation of Modern Pathology to Dentistry." He did this with diffidence, but this disappeared "before the opportunity to pay tribute to a man who has ennobled his profession and honored his country in foreign lands, demonstrating, as he has done, the truth of the relation of modern pathology to scientific dentistry.

"Thirty years ago there worked a giant intellect in a small laboratory in Paris. There Pasteur, with the world of science against him, discovered the influence of micro-organisms upon organic matter. Then he demonstrated that the true nature of fermentation and putrefaction was due to the influence of micro-organisms. Lister's practical mind used the methods of Pasteur for the elimination of bacteria, and gave to the world the blessing known as antiseptic surgery. There are processes of putrefaction in the mouth due to the invasion of particles of food by micro-organic growth, producing a corrosive substance that gradually impairs the enamel and destroys the dentine. To the honor of Professor Miller be it said, he was the first to pay attention to this process, and to discover the fact that the cause was lactic acid, and which, joined with the lime, produces lactate of lime."

In closing, he said, "There is the science and there is the art of dentistry, and he is the most eminent who, through a full measure of science, has acquired the highest development of the art. Therefore, Professor Miller, that your life-work should find its crown of glory, let us drink, while every goblet foams, the wish that dentistry, now raised to the dignity of a science, may soon enter the same career as her sister specialty, and work under that banner of medicine whose motto is 'the alleviation of human suffering.'"

TOAST: *The Bond of Union between Dentistry and Medicine.*

Professor Harrison Allen remarked, in allusion to those of the President, "I began my career as a dentist, but the charms of medicine soon lured me away, but in after years I was again in a position particularly congenial to me,—that of teaching anatomy to dental students at the Philadelphia Dental College." He then alluded to a valedictory in which he criticised dentists and their work in the line of investigation, and presumed that the irritation that this caused had been forgotten, from the fact that he was called upon to speak to the toast, "The Bond of Union between Dentistry and Medicine." "What about this bond of union? It seems from this evening's proceedings there is no bond, because it is one thing. If there is no bond, there is no use talking about it; but if there is an idea that there is a bond, and that it unites two things, not entirely similar, it opens a subject not touched upon, and upon which I will not enter at length, even were I capable of doing it justice."

Dr. Allen then illustrated the union of Surgeon, Barber, and Dentist, by a sign he met with in a town in which he was a temporary resident during the summer, and closed his very interesting remarks as follows:

"While I have been sitting here I have been reflecting upon another subject,—the improvements in medicine. We, the English-speaking people, can claim much of this work. For example, the great Sydenham made the first scientific system of medicine, and I recall the anatomical and pathological studies in which Hunter stands first. Cannot we congratulate ourselves upon another fact when we come down to dentistry: we have one young Englishman—the younger Tomes—and Professor Miller on the continent to represent us. So many expressions have been and can be made to show that dentistry was at one time on a lower plane than medicine, but I think both sciences may be compared to a beleaguered city awaiting supplies, or to the conundrum, 'Why is a beleaguered city like a new-born infant?' and you will remember the answer is that 'the long expected *succour* has at length arrived.'"

The President called upon the chairman of the committee for some volunteer remarks.

*Dr. Edw. C. Kirk.*—"In responding to this call of the toastmaster, I find myself confronted with a dilemma," and then quoted an anecdote illustrative of the fact that "all the best words had been picked out," leaving nothing for him to say. "I feel, however, that I should like to explain, on behalf of the committee, the gratification which they feel in having had an opportunity to present before an audience of this character a dental ideal towards which many of our profession are striving, an ideal which has been so largely realized in the work of our distinguished guest. Now, as it has not been spoken of definitely, I should like to call your attention to a short epitome of what that work has been. In the fall of 1877 he came to Philadelphia and matriculated at the Pennsylvania College of Dental Surgery, completing his course in the Dental Department of the University of Pennsylvania. Returning to Europe in 1879, he began the practice of dentistry, and at the same time pursued his studies at the University of Berlin. The result of his labors there were published in a series of papers, beginning with one on 'The Agency of Micro-Organisms in Caries of the Human Teeth,' published simultaneously in Kleb's *Archives*, and the *Dental Cosmos.* This was followed by some thirty communications in the same line of research, the most important of which are:

"1. A series of papers on 'Fermentation in the Human Mouth.'

"2. A series entitled 'Biological Studies on the Fungi of the Human Mouth.'

"3. 'Micro-Organisms of the Alimentary Tract.'

"4. 'Wörtenbuch der Bacterien Kunde.'

"5. A larger work,—'Die Mikro-Organismen der Mundhöle.'

"This latter work is now ready to appear in English. When his first thesis was published, in which was established the nature of dental caries, and in which he had proved to the point of demonstration the exact nature of the process, and while the dental profession of the two continents applauded and all were astonished, there were a few, however, who were not surprised. These were the men who had sat on the college benches with him, and were familiar with his qualities and methods of work while there. There is one peculiarity illustrated in his method of work which calls to the mind the evidence given in a certain murder case. The witness stated that he was thirty-two feet eleven inches from the defendant when he fired the fatal shot. During the cross-examination the attorney said, 'Now, you have testified that you were thirty-two feet eleven inches away; how do you know that was the exact distance?' 'Because,' replied the witness, 'at the time I thought, perhaps, some fool might ask that question, so I went and measured it.' Now, Professor Miller has had from the beginning the habit of measuring things with accuracy and precision in his work, and it is this as much as anything which gives value to his results, which are not speculative in character, but thoroughly practical and clearly scientific, as has been so well shown in his work on dental caries. Now we feel we are not here to honor Dr. Miller in the ordinary sense, for a man with such a record needs no greater honor, but we are here to show our appreciation of his achievements. We appreciate him first as an American who has made a record in Europe, and compelled recognition in a city where intellectual advancement and learning are of the highest order; we appreciate and are grateful to him for the impetus which he has given to the cause of scientific dentistry, and there are not a few of us who feel proud that on the roster of the first class which went out from the Dental Department of the University of Pennsylvania stands the name of W. B. Miller, of Ohio."

Dr. H. C. Wood was called upon; but he declined at that late hour to add anything except to relate an anecdote illustrative of his position.

The happy introductions of the toast-master of the evening, Professor E. T. Darby, have necessarily been omitted in the report; but they added very much to the interest of the evening.

The Committee of Arrangement, upon whom the burden of this reception fell, and to whom great credit is due for the satisfactory results, are as follows: Edward C. Kirk, chairman; S. H. Guilford, Daniel Neall McQuillen, Alonzo Boice, John D. Thomas, secretary.

### DEATHS FROM CHLOROFORM IN NEW SOUTH WALES.

PARTICULARS of deaths while under effects of chloroform in New South Wales, from the year 1885 to date:

| Date of death. | Date of inquest. | Name of deceased. | Age. | Locality of death. | Anæsthetic used. |
|---|---|---|---|---|---|
| 1885. | 1885. | | | | |
| June 17 | June 18 | James Kelly | 27 | Gulgong Hospital | Chloroform |
| July 30 | Aug. 1, 4 | Olivia Green | 37 | Sydney Hospital | Chloroform |
| 1886. | 1886. | | | | |
| Feb. 16 | Feb. 17 | Philip Norris | 13 | Albury Hospital | Chloroform |
| July 13 | July 13 | William Lancaster | 45-50 | St. Vincent's Hospital | Chloroform |
| 1887. | 1887. | | | | |
| March 5 | March 7 | Walter Charles Wagstaff | 33 | Sydney Hospital | Chloroform |
| April 28 | April 28 | Alexander De Berg | ... | Wagga Hospital | Chloroform |
| May 10 | May 11 | George Merritt | 30 | Newcastle Hospital | Chloroform |
| Aug. 22 | Aug. 23 | Mary Gilt | 36 | Sydney Hospital | Chloroform |
| Aug. 25 | Aug. 25 | John David Rhodes | 35 | Goulburn Hospital | Chloroform |
| Oct. 22 | Oct. 24 | Julia Constance Campbell | 34 | Dr. R. Wood's Hospital, Syd. | Chloroform |
| 1888. | 1888. | | | | |
| Feb. 29 | March 3 | Mary Ann Williams | 49 | Prince Alfred Hospital | Chloroform |
| April 1 | April 3 | Lee Yum Kay | 54 | Sydney Hospital | A.-C.-E. mixture |
| 1889. | 1889. | | | | |
| Feb. 6 | Feb. 8 | James Maxton | 30 | Prince Alfred Hospital | Methylene |
| June 3 | June 5 | Charles Edward Christian | 44 | Prince Alfred Hospital | Chloroform |
| Nov. 19 | Nov. 22 | Henry Nutall | 37 | Gladesville Lunatic Asylum | Chloroform |
| 1890. | 1890. | | | | |
| ...... | Jan. 3 | Peter Donnelly | 67 | Tamworth Hospital | A.-C.-E. mixture |
| Feb. 15 | Feb. 15 | Louis Smith | 22 | Albury Hospital | Chloroform |
| April 23 | April 24 | George Galvin | 14 | Prince Alfred Hospital | Chloroform |
| May 15 | May 17 | Charles Segol | 27 | Prince Alfred Hospital | Chloroform |
| May 19 | May 21 | Catherine Gollan | 7 | Prince Alfred Hospital | Chloroform |

